# Vehicle Classification Based on FBG Sensor Arrays Using Neural Networks

**DOI:** 10.3390/s20164472

**Published:** 2020-08-10

**Authors:** Michal Frniak, Miroslav Markovic, Patrik Kamencay, Jozef Dubovan, Miroslav Benco, Milan Dado

**Affiliations:** Faculty of Electrical Engineering and Information Technology, University of Zilina, 01026 Zilina, Slovakia; michal.frniak@uniza.sk (M.F.); miroslav.markovic@uniza.sk (M.M.); jozef.dubovan@uniza.sk (J.D.); miroslav.benco@uniza.sk (M.B.); milan.dado@uniza.sk (M.D.)

**Keywords:** vehicle classification, FBG, artificial intelligence, smart sensors

## Abstract

This article is focused on the automatic classification of passing vehicles through an experimental platform using optical sensor arrays. The amount of data generated from various sensor systems is growing proportionally every year. Therefore, it is necessary to look for more progressive solutions to these problems. Methods of implementing artificial intelligence are becoming a new trend in this area. At first, an experimental platform with two separate groups of fiber Bragg grating sensor arrays (horizontally and vertically oriented) installed into the top pavement layers was created. Interrogators were connected to sensor arrays to measure pavement deformation caused by vehicles passing over the pavement. Next, neural networks for visual classification with a closed-circuit television camera to separate vehicles into different classes were used. This classification was used for the verification of measured and analyzed data from sensor arrays. The newly proposed neural network for vehicle classification from the sensor array dataset was created. From the obtained experimental results, it is evident that our proposed neural network was capable of separating trucks from other vehicles, with an accuracy of 94.9%, and classifying vehicles into three different classes, with an accuracy of 70.8%. Based on the experimental results, extending sensor arrays as described in the last part of the paper is recommended.

## 1. Introduction

The issue of traffic monitoring and management has arisen due to a growing number of personal vehicles, trucks, and other types of vehicles. Due to existing road capacities being based on historic designs, the condition of these road communications deteriorates with a lack of growing financial investment to maintain and expand the road network. With these requirements, vehicle visual identification is not sufficient for traffic management and the prediction of the future state of traffic and road conditions. For this purpose, existing monitoring areas are being innovated with new sensor platforms, not only for the statistical purpose of monitoring areas. Additional information such as traffic density, vehicle weight distribution, overweight vehicles, and trucks could be included in automatic warning systems for the prediction of possible critical traffic situations. There are several technological approaches based on different principles. Each of them has various advantages and disadvantages, such as operating duration, traffic density, meteorological condition limits, resistance to chemical and mechanical damage from maintenance vehicles, etc.

All motor vehicles are classified into 11 base classes by current legislation in the states of the central European Union. Meanwhile, according to the Federal Highway Administration under the United States Department of Transportation, there are even 13 classes. These classes consist of personal vehicles, trucks, technical vehicles, public transport vehicles, and their subclasses. For decades, the only sufficient method to classify vehicles was by visual recognition. This method was strongly limited by meteorological conditions. In the last two decades, several different technical designs for classifying vehicles without a visual part of classifications have been proposed. At first, based on metallic vehicle chassis and axle parts, there were designs to measure magnetic field parameters of crossing vehicles. This included inductive loops or anisotropic magneto-resistive sensors built into the road pavement [1,2,3,4]. These technological designs achieved accurate results for specific vehicle classes with magnetic signatures. A different approach was by vehicle weight signature. Technological solutions based on piezoelectric sensors [5] and bending plate sensors are widely used in road traffic monitoring and vehicle measurements [6]. There were also experimental solutions such as the usage of hydro-electric sensors [7] with a bending metal plate at the top of the vessel filled with a specific liquid. Weigh-in-motion technologies measuring specific parameters such as the weight signature could be used, as well as other technologies including fiber optic sensors [8], wireless vibration sensors [9], or using embedded strain gauge sensors [10]. As an additional capability, this could be measured by smart pavements based on conductive cementitious materials [11]. Optic sensors based on Fiber Bragg Grating (FBG) were also successfully tested on different types of transport, such as railways. It was in Naples in Italy where this type of sensor was used for speed and weight measurements with the detection of train wheel abrasions as additional information for transport safety [12].

Vehicle classification and the measurement of vehicle parameters, such as weigh-in-motion, were the aim of several international research projects. The weighing-in-motion of road vehicles was a research aim in the European research project COST323 over two decades ago [13]. In the last decade, research ideas relating to infrastructure monitoring including road traffic have been studied, e.g., by COST projects TU1402 for structural health monitoring and TU1406 for roadway bridges [14,15,16].

Optical fiber sensors are becoming a very important part of smart Internet of Things (IoT) infrastructures, also on roads and highways. They can additionally perform different functions in critical infrastructure protection and monitoring. There is a broad spectrum of technological solutions of fiber optic sensors and optical sensors systems. For our investigation, we used the FBG sensor network built into the entry road into the campus of our university. Fiber Bragg Grating (FBG) sensors are classified as passive optical fiber components that are compatible with existing types of telecommunication fiber systems and can operate directly with incident light (most commonly in the 1550 nm range). Thus, they can be directly incorporated into the optical transmission chain. The fundamental principle on which the FBG work is based is Fresnel diffraction and interference. The propagating optical field may be refracted or reflected at the interface in the transmission medium with different refractive indices. The FBG operates as a light reflector for a specific (desired) spectrum of wavelengths, to ensure that the phase-matching condition is met. Other (undesirable) wavelengths are only slightly influenced by the Bragg grating [17,18,19].

In recent years, the different Convolutional Neural Network (CNN) architectures [20,21,22] applied to image processing constitute the current dominant computer vision theory, especially in tasks such as image classification (vehicle classification). The main goal of these networks is to transform the input image layer-by-layer from the input image to the final class scores. The input image is processed by a series of convolution layers with filters (kernels), max pooling layers, and Fully Connected (FC) layers. Finally, the activation function, such as softmax or sigmoid to classify the outputs (small cars, sedans, crossovers, family vans, or trucks), is used. In our case, the AlexNet [20] and GoogLeNet [23] convolutional neural networks were chosen. The basic architecture of the AlexNet consists of some convolutional layers (five layers), followed by max pooling layers, FC layers (three layers), and a softmax layer [20,24,25]. On the other hand, the architecture of GoogLeNet consists of 22 layers (nine inception modules). The main motivation for the inception modules’ (layers’) creation is to make a deeper CNN network so that highly accurate results could be achieved [23,26,27]. For vehicle classification, several works using deep learning and convolutional neural networks were described in [20].

The aim of this article is vehicle classification with FBG sensor arrays using artificial intelligence from partial records. The proposed neural network was trained using a dataset with a lack of information on the vehicle’s speed, which we created by visual recognition of the vehicle passing through our testing platform. The majority of recorded vehicles were detected only through their left wheels, which reduces records from a 3D vehicle surface to one line of deformation. These records simulated situations where the vehicle’s driver tried to avoid detection with a changed trajectory through the roadside or an emergency line without visual recognition.

## 2. Materials and Methods

The main goal of the research is the use of optical sensor networks for the classification of passing vehicles through a test platform based on neural networks for car recognition using an industrial camera. For this purpose, a test platform was built, which is described in Section 2.1.

### 2.1. Experimental Platform

**Figure 1:**
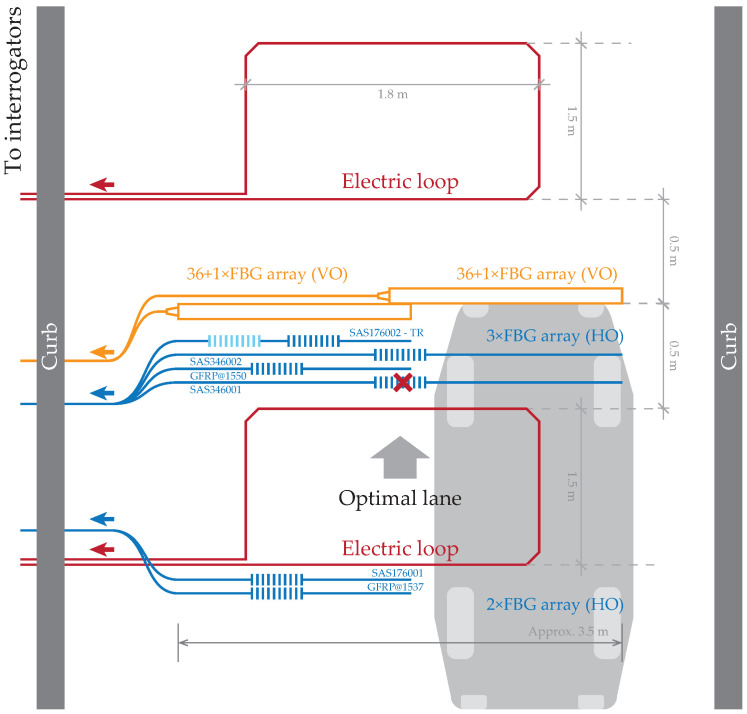
Real test platform scheme with multiple Fiber Bragg Grating (FBG) sensors. Some are connected as an FBG sensor array. The red cross indicates a dysfunctional FBG sensor (destroyed when the test platform was created). This test platform was built on the road into the university campus.

The test platform for the measurement of additional vehicle characteristics is located at the University of Zilina campus on the entry road to the main parking lot. This monitoring area consists of several sensor arrays based on two technological applications of FBG sensors. All these sensors are built in the 2nd asphalt pavement layer covered with a top asphalt layer with a height of 6 cm above the sensors. Two electric loops were installed for the initialization of measurements, but the main goal was to use only optic-based sensors as FBGs. Those were realized in two different placements and numbers, as shown in Figure 1.

#### 2.1.1. Vertically Oriented FBG Sensors

**Figure 2:**
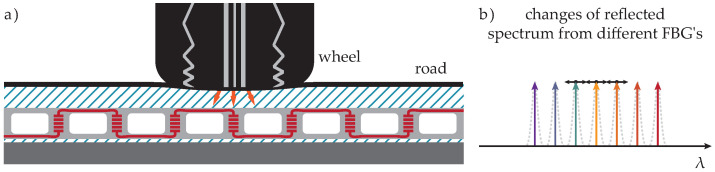
(**a**) Wheel pressure is applied to vertically oriented FGB sensors; (**b**) reflected optical spectrum shift is given by pressure change (every FBG reflects light on the other’scentral wavelength in idle status—the FBG’s position is also known).

The 1st type of FBG was attached vertically on a perforated aluminum chassis with approximately a 10 cm distance between these Vertically Oriented (VO) sensors (orange sensors in Figure 1) positioned orthogonally to the direction of the vehicle, as shown in Figure 2. Based on the configuration of these sensors and their placement, there are several limitations. One of them is the distance between vertical FBG sensors. Each vehicle’s wheel is captured in a range from 3 to 4 vertical FBG sensors. Due to the construction of the aluminum chassis with these sensors in a partially liquid material such as asphalt, it is problematic to determine wheel width. This is a necessary parameter for calculating the weight distribution area through measuring the wheel and accurately determining the vehicle class.

#### 2.1.2. Horizontally Oriented FBG Sensors

**Figure 3:**
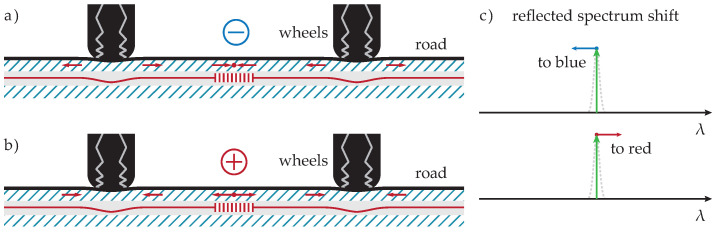
Illustration of some scenarios of wheel pressure to horizontally oriented FGB sensors (**a**) when the wheel’s pressure in the horizontal line is negative (compressive stress) or (**b**) positive (tensile stress), (**c**) and the appropriate reflected light spectrum change in wavelength.

The second type of FBG sensor was horizontally placed orthogonally (blue sensors in Figure 1) at different distances from vertical FBG sensors in the direction of the vehicle. Horizontally Oriented (HO) FBG sensors were installed with two different active lengths of sensors (measured on the whole fiber length using one FBG sensor). The first sensor had a length of 3460 mm, and the second had a length of 1760 mm. One of the optical fibers with shorter sensors contained another FBG for temperature compensation. Both horizontal sensor lengths had a passive length of 300 mm and an operative temperature range from −40 to +80 °C. All horizontal sensors were attached to the bottom asphalt layer by asphalt glue. This allowed for the measurement of exact flexibility and strength changes of the top asphalt layers during the measurements of overpassing vehicles. Due to the vehicle wheel trajectory over those sensors and their type, we observed both compression and tension, as shown in Figure 3.

#### 2.1.3. Measurement Units

Each set of measurement data was from the FBG sensor arrays consisting of 2 lines of 36 vertical sensors orthogonal to the vehicle direction and 2 sensors for the temperature compensation of the vertical sensors. From the horizontal FBG sensors, there were 3 horizontal sensors at a different level. Two of those sensors had an active length of 1760 mm, and one had a length of 3460 mm. One fiber with a shorter length contained an FBG sensor for temperature compensation created for different wavelengths. The sampling rate of the two interrogators connected to the FBG sensor arrays was 500 samples/s.

**Figure 4:**
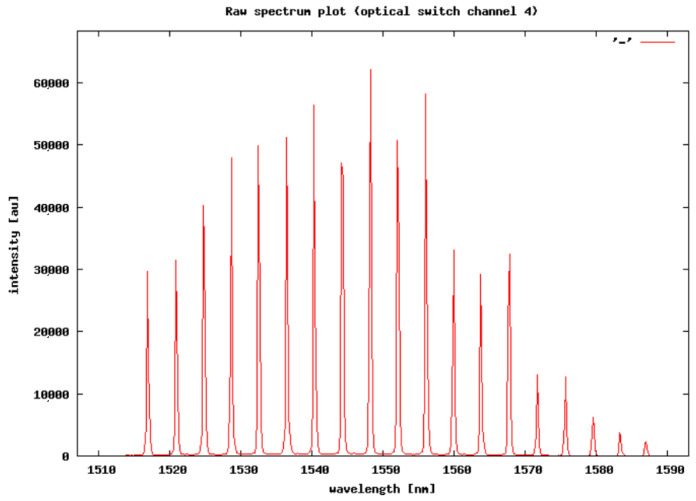
Time sample of the reflected optical spectrum from the FBG array received by the interrogator.

Output matrix data of each measurement had 2000 time samples (4 s) of the 34 vertically oriented FBG sensors used. This output matrix was extended by measurements from 4 horizontally oriented FBG sensors with a dimension of 2000 time samples (4 s). We used only 34 of 36 vertical FBG sensors because the last 2 peaks of reflected intensity on specific wavelengths were too low for processing in the interrogator, and this caused problems with measured data consistency, as shown in Figure 4.

The 1st peak value of the FBG sensor, set at 1517 nm, was dedicated to temperature compensation. The last 2 unused vertical FBG sensors were preset at wavelengths of 1583.74 and 1587.68 nm. Both matrices for 2000 measurements were synchronized into the same time range. This format and size of data were applicable only in one direction of the vehicles due to the position of each sensor array.

### 2.2. Proposed Methodology

**Figure 5:**
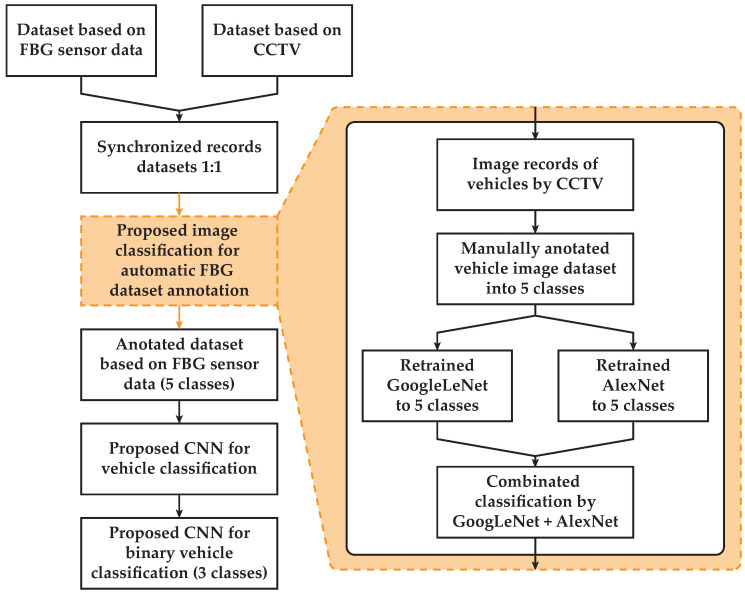
Block diagram of the proposed methodology.

The block diagram of the proposed methodology is shown in Figure 5. Firstly, datasets based on FBG sensor data and Closed-Circuit Television (CCTV) were created. Next, the modified neural networks for visual classification using a CCTV camera system for FBG dataset annotation were used. This classification was used for the verification of measured and analyzed data from the sensor arrays. Finally, the newly proposed neural network for vehicle classification from the sensor array dataset was created.

Two separate datasets were created. Firstly, an image dataset based on CCTV was created for the acceleration of the automatized learning process for vehicle classification based on FBG sensor data. Secondly, a dataset based on FBG sensor data was created for final vehicle classification by the proposed CNN.

#### 2.2.1. Dataset Based on FBG Sensor Data

**Figure 6:**
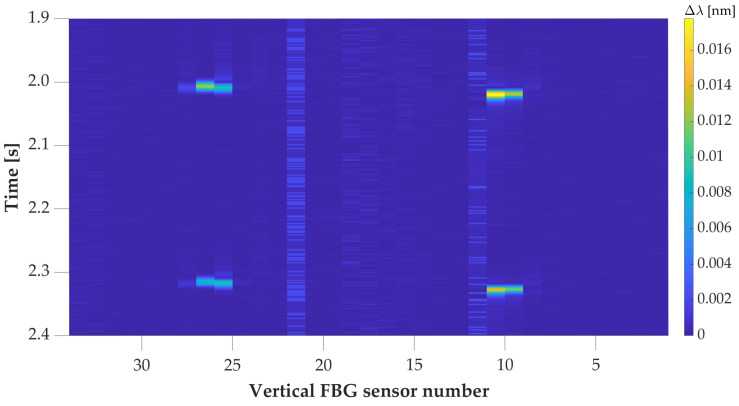
Record detail of the pressure map for a vehicle with an optimal line. The colormap represents the values of the wavelength change of the reflected optical spectrum by FBG in nm.

Each vehicle’s record from the test platform was created with a matrix from vertical FBG sensors with a size of 2000 measurements by 34 sensors. With a sampling rate of 500 samples/s, this represents a period of 4 s per each vehicle. The record detail of the full pressure map of the vehicle with a wheelbase of 2570 mm is presented in Figure 6.

**Figure 7:**
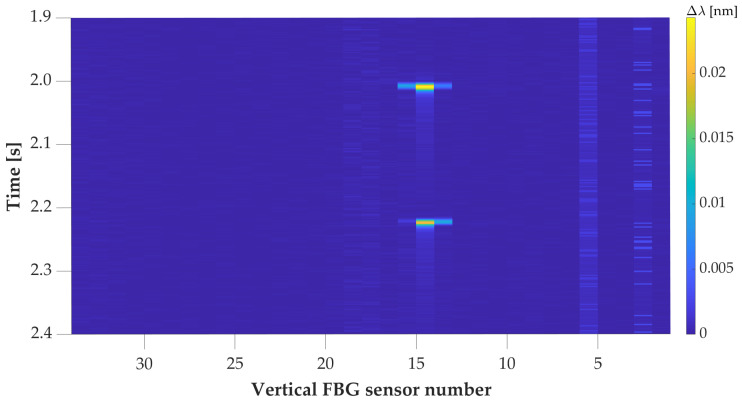
Record detail of the pressure map for the overpassing vehicle with left wheels. The colormap represents the values of the wavelength change of the reflected optical spectrum by FBG in nm.

The shift in samples for each axle between the wheels in Figure 6 is caused by the installation shift of aluminum strips for vertical FBG sensors, shown in Figure 2 with orange color. The partial pressure map (only left wheels) of the vehicle with a wheelbase of 2511 mm is in Figure 7. Both vehicle’s details show the detection of the 1st axle at time position 2 s. This was based on two way detection.

**Figure 8:**
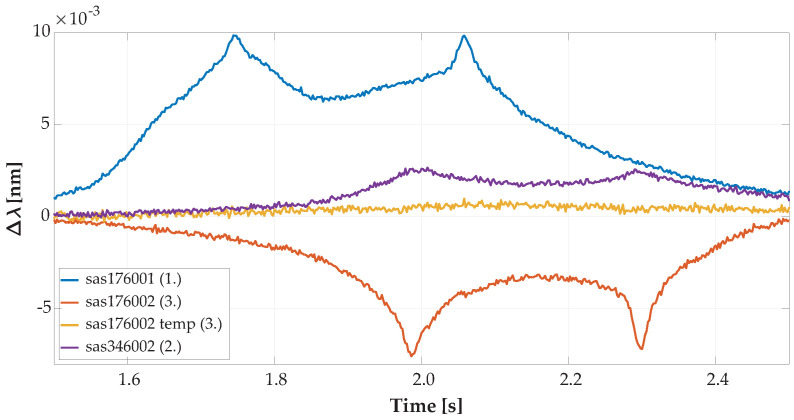
Record detail of axle detection from horizontal FBG sensors from the overpassing vehicle with an optimal traffic line as reflected in the optical spectrum wavelength change detected by the FBGs.

**Figure 9:**
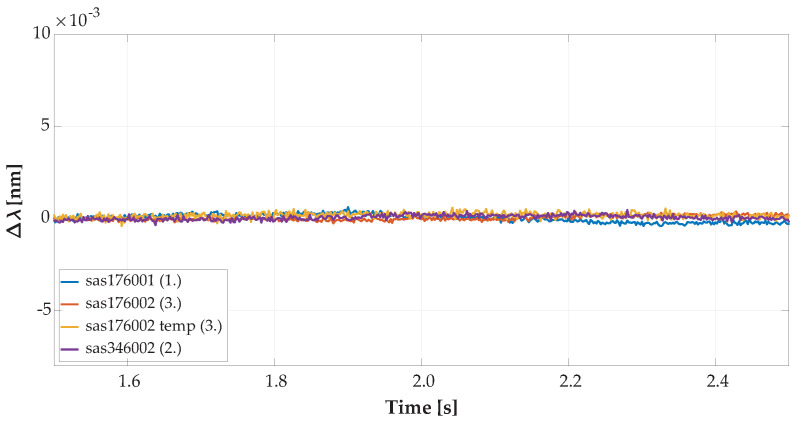
Record detail of axle detection from horizontal FBG sensors from the overpassing vehicle with a non-optimal traffic line as reflected in the optical spectrum wavelength change detected by the FBGs.

For speed determination without information on the specific wheelbase of the vehicle from visual recognition, there were built-in horizontal FBG sensors of 2 lengths. Those sensors were placed asymmetrically towards the left side of the road. Record details from the overpassing vehicle recognized by both lines are shown below in Figure 8 and the overpassing vehicle recognized by one line in Figure 9.

Figure 8 is a record detail of the same vehicle’s record as shown in Figure 6. A vehicle with the optimal line was captured with vertical and horizontal sensors; thus, we were able to determine vehicle speed and wheelbase distances. In Figure 7 and Figure 9 is shown the same overpassing vehicle recognized by only one line of wheels by vertically oriented FBG sensors.

Records with only one line (footprint) of wheels of the vehicle recognized by vertically oriented FBG sensors, and those vehicles that were not recognized by horizontally oriented FBG sensors and measured data seem to be akin to a Nothing-on-Road state (NoR).

For the simplification of vehicle detection, we summed all wavelength shifts of all vertical FBG sensors per each timestamp. The summed wavelength shift for all *k* sensors in specific time $t_{n}$ was compared with the summed wavelength shift for all *k* sensors in previous time $t_{n-i}$. Reference value $\Delta \lambda_{R}$ was added to this value, which corresponds to the minimum recorded pressure on the sensors from one vehicle’s wheel detection. The reference value of $\Delta \lambda_{R}$ for the 1st axle detection was 0.015 nm with an air temperature over the test platform in the range from 15 to 30 °C. The equation for the 1st axle’s detection is:

(1) $$\sum_{k}\left|\Delta \lambda_{k,t_{n-i}}\right|+\Delta \lambda_{R}\leq \sum_{k}\left|\Delta \lambda_{k,t_{n}}\right|.$$

**Figure 10:**
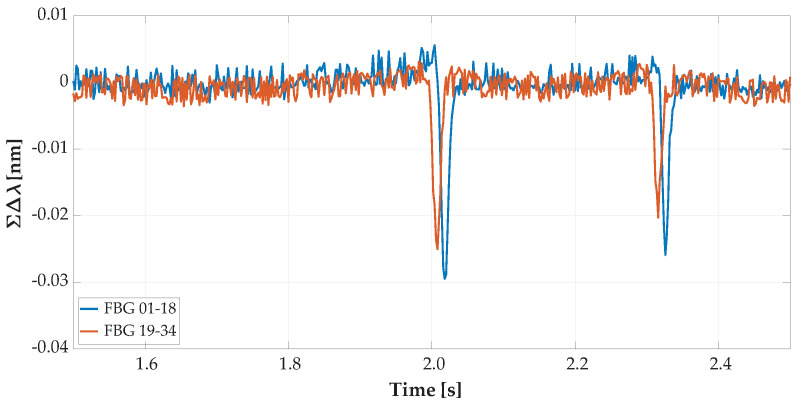
Record detail of the summary values of the wavelength changes (of the reflected optical spectrum by FBG) shifted by Nothing-on-Road (NoR) values.

The record details of the summed values per 2 strips with vertical FBG sensors shifted by NoR values are shown below in Figure 10. The right wheels of vehicles shown by the blue curve for summed sensors with Positions 1 to 18 were detected. The left vehicle wheels are shown by the orange curve for summed sensors with Positions 19 to 34 by the left strip with vertical FBG sensors. The record detail shown is for the same vehicle as in Figure 6 and Figure 8.

**Figure 11:**
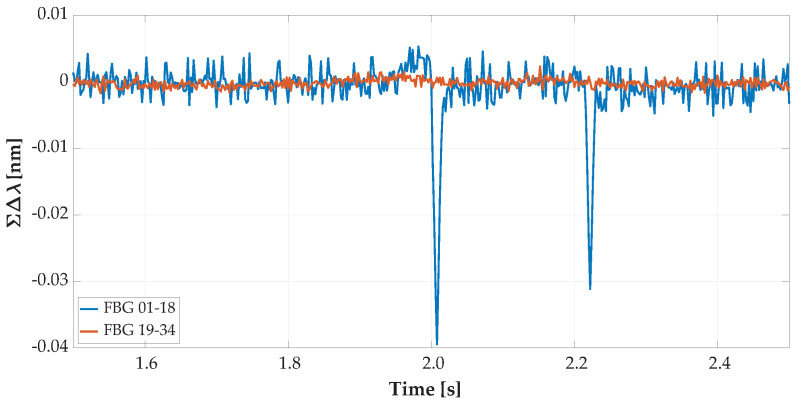
Record details of the summed wavelength shifts (of the reflected optical spectrum) from the vertical FBG sensors of the overpassing vehicle recognized only by the left wheels.

On the graph of the overpassing vehicle recognized only by one line of vehicle wheels in Figure 11, there was a partial record with no detection of the vehicle’s right wheels. Only left wheels were detected by the sensors in Positions 1 to 18 with a blue curve.

Figure 7, Figure 9 and Figure 11 depict the same partially recognized vehicle, where it was not possible to determine the vehicle’s speed and wheelbase distances from the minimal two lines of the FBG sensors. This information could only be used in combination with visual identification of the vehicle’s model with technical parameters.

#### 2.2.2. Dataset Based on CCTV

**Figure 12:**
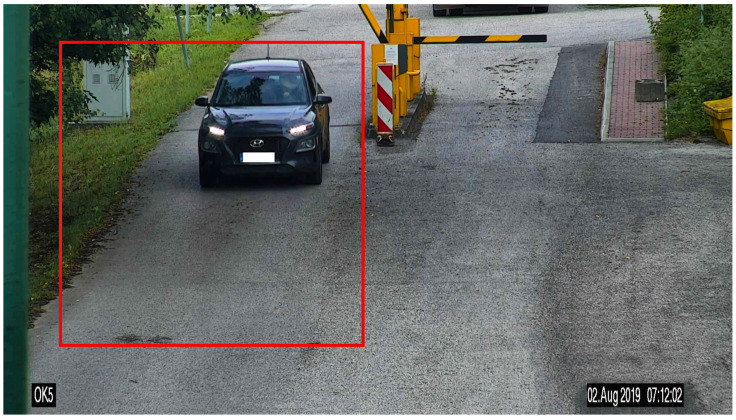
Entry ramp view from CCTV with the area of interest (red rectangle with a resolution of 800 × 800 px) with a timestamp.

Our test platform is incapable of accurately determining wheel width and other additional parameters based on it. For this reason, we decided to define each vehicle class by wheelbase and weight ranges in combination with visual recognition. For this, we used security CCTV monitoring the entry ramp used to access the road with the testing platform. This entry ramp serves as a measurement separator in the direction of monitored vehicles, as shown in Figure 12.

The input images from CCTV were at a resolution of 1920 × 1080 px. The area of interest, with an image size of 800 × 800 px (red rectangle), is shown in Figure 12.

#### 2.2.3. Synchronized Records’ Datasets

All vehicles were monitored with CCTV and measured using FBG sensor arrays for 1 month. Per each overpassing vehicle’s record, there was 1 synchronized vehicle image. These images were classified by 2 CNNs for image classification, validated as shown in Figure 5, and integrated with records from FBG sensor arrays. Those records were impossible to classify only from vertically oriented FBG sensor arrays without image classification. For the next vehicle’s classification using FBG sensors, there were only relevant data from the chassis of vertical FBG sensors from Positions 1 to 18.

#### 2.2.4. Proposed Image Classification for Automatic FBG Dataset Annotation

For the visual verification of the 5 determined classes, we tested the dataset on 3 different CNNs in the MATLAB^®^ workspace in Version 2019b. We decided to use AlexNet [12], GoogLeNet [13], and ResNet-50 [28,29]. Each pre-trained network was modified in the final layers for specific class number outputs.

**Figure 13:**
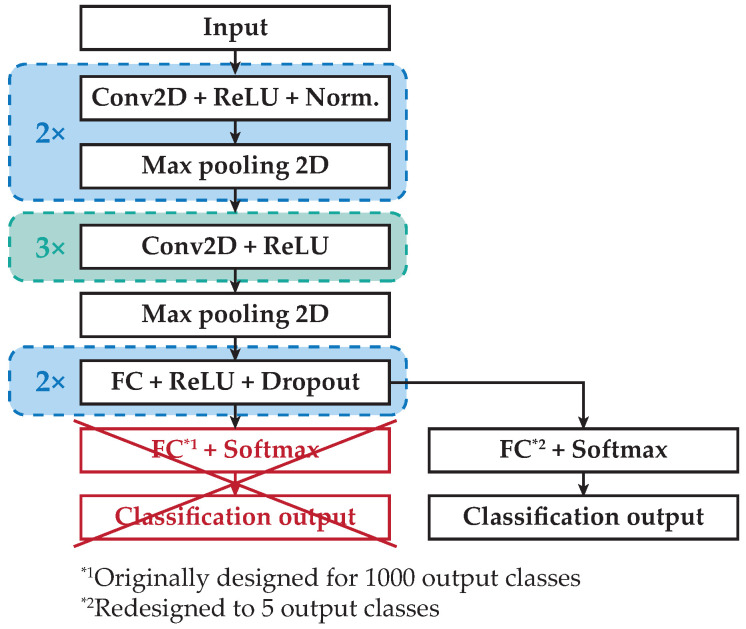
Architecture modification of the pretrained AlexNet.

**Figure 14:**
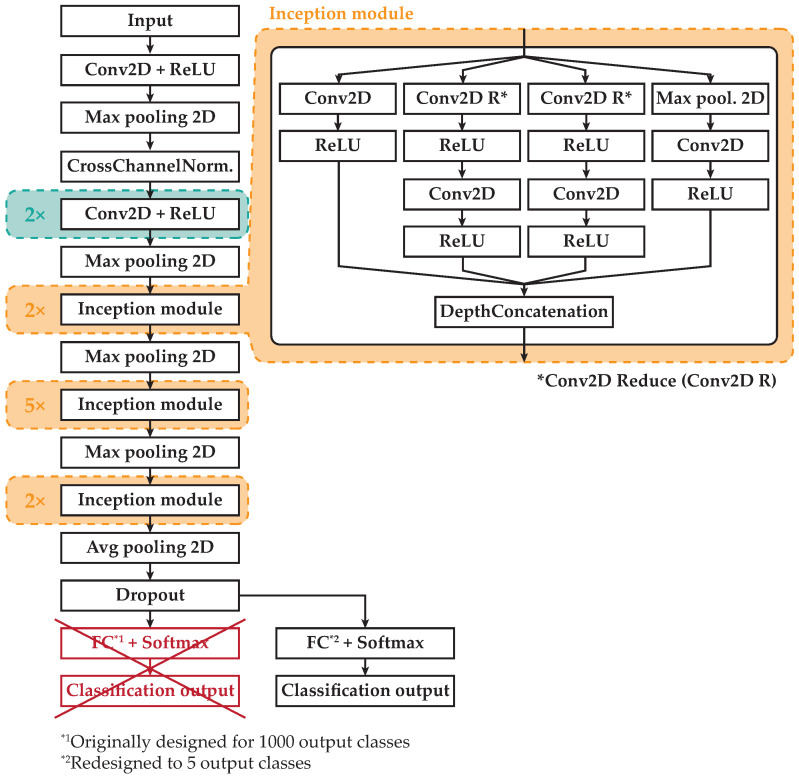
Architecture modification of the pretrained GoogLeNet.

The architecture modification of the pretrained CNN AlexNet from 1000 classes to 5 classes is shown in Figure 13. The modification of pretrained Directed Acyclic Graph (DAG) CNN GoogLeNet with the same number of pretrained classes as AlexNet to 5 classes is shown in Figure 14.

The training phase consisted of 650 vehicle images for each class. The test phase consisted of a minimum of 100 vehicle images for each class. Those images were next resized to the necessary input size to each CNN [20,23].

For this reason, we decided to create 5 vehicle classes. The 1st class was small cars with hatchback bodyworks with a weight up to 1.5 t and up to a 2650 mm axle spacing. The 2nd class was vehicles such as sedans and their long versions or combo bodyworks. The 3rd class was vehicles with crossover bodyworks and Sports Utility Vehicles (SUV). The 4th class was utility vehicles and family vans weighing up to approximately 2.5 t. The last class was vans, trucks, and vehicles with more than 2 axles. Motorcycles were excluded from the classification. These 5 classes were also determined based on the composition of the vehicles (see Table 1) and their count crossing the campus area with a test platform.

Each CNN was retrained 5 times for the 6 epochs achieved, in equal conditions, with an accuracy of over 90% in the tested dataset. One epoch represents the processing of all training samples. After that, training samples were shuffled for the next epoch. Those CNNs were supervised and retrained by using a Graphic Processor Unit (GPU) with only 2 GB GDDR5 memory in previous research. The accuracy of the created dataset was enough for our purpose of classifying data from FBG sensor arrays [30].

Thus, the retrained CNNs for image classification were prepared to classify vehicle records from FBG arrays using the visual part of the records. Each record from the arrays was synchronized with 1 image from the industrial camera taking into consideration the distance between the entry ramp and the measured sensory area. The synchronized image dataset was divided into 2 identical datasets with resolutions of 224 × 224 px for GoogLeNet and 227 × 227 px for AlexNet classification. In 77.26% of the images, both CNNs were consistent. The accuracy of the CNNs used for visual classification is shown in Table 2. Other images were manually verified and included in the correct classes.

#### 2.2.5. Annotated Dataset Based on FBG Sensor Data

The prepared dataset consisted of 5965 vehicle records recognized with only one line using vertically oriented FBG sensors divided into 5 classes. This dataset did not contain vehicle speed, wheelbase, or wheel size information. For simple classification, a neural network was created in the Integrated Development Environment (IDE) MATLAB^®^ 2020a for image input in the Tagged Image File Format (TIFF) with a resolution of 600 × 5 px (600 time samples × 5 vertically oriented FBG sensors). These data were normalized into a range from 0 to 1 with eight decimal precision and were saved in TIFF format per each partial record without data compression.

#### 2.2.6. Proposed CNN for Vehicle Classification

The structure of the CNN created is in Table 3 below. The CNN was tested for various dataset interclass combinations. Due to wheelbases and the speed of overpassing vehicles, up to 600 samples per record (1.2 s, see Figure 6) were recorded for all vehicles, trucks included. Most of the small vehicles’ last wheel was on average recorded up to a time sample of 200 records (0.4 s) for speeds under 50 km/h and the last wheel up to a time sample of 500 records (1 s) per all vertical sensors with speeds under 10 km/h.

For that reason, the 1st 2D convolution layer was set to filter sizes from 300 to 4, covering at least a wheel per record in the 1st layer. Enlarging the filter size on the 1st layer during training did not show any improvements. After a 2D max pooling layer, a last 2D convolution layer was added with a filter size of 50 to 2. This design showed the best-achieved results for binary classification on our prepared dataset. After the last convolution laser, there was a fully connected layer with the softmax function to assign the result to only one of all output classes based on an overall number of trained classes.

For training purposes, there were 800 vehicle samples separated from the first 4 classes and 400 samples from the last truck class. Those samples were divided by a ratio of 9:1 for the input training set and validation set during training.

## 3. Experimental Results

For all CNNs, the training had the same option setup as training for 200 epochs with the batch size set to twenty. On the main diagonal in the confusion matrix, correctly classified vehicles of all tested vehicles are shown in Table 4. For the first class (hatchback class), forty-nine-point-six percent of vehicles were correctly classified (valid column), as shown in Table 5. For the second class (combo/sedan class), twelve-point-point-eight percent of vehicles were correctly classified. For the third class (SUV class), fifty-six-point-three percent of vehicles were correctly classified. For the fourth class (MPV/minivan class), twenty-six-point-eight percent of vehicles were correctly classified. For the last class (van/truck class), sixty-two percent of vehicles were correctly classified. An overall accuracy of 28.9% for all tested vehicles using the proposed CNN was achieved. Due to the classification into five classes and their similarities, a validation accuracy of only 28.9% was achieved.

The proposed CNN was modified to three classes for better spatial separation of classes. On the main diagonal in the confusion matrix, correctly classified vehicles of all tested vehicles are shown in Table 5. For the first class (hatchback class), seventy-four-point-three percent of vehicles were correctly classified (valid column), as shown in Table 5. For the second class (SUV class), thirty-seven-point-eight percent of vehicles were correctly classified. For the third class (van/truck class), seventy-eight-point-nine percent of vehicles were correctly classified. An overall accuracy of 60.0% of all tested vehicles using the proposed CNN was achieved.

The proposed CNN was modified for classification between two classes (hatchback class to van/truck class). An overall accuracy of 92.7% for both tested vehicle classes, as shown in Table 6, using the proposed CNN was achieved.

### Proposed CNN for Binary Vehicle Classification

**Figure 15:**
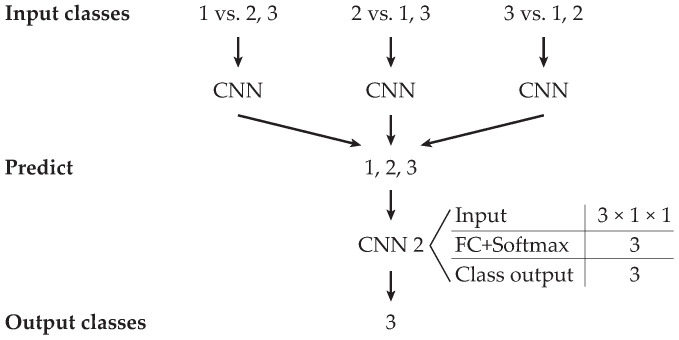
Process of the vehicle classification of overpassing vehicles.

For the improvement of the achieved validation for three classes in the combination of binary classification, we used the designed CNN for classification of three variations of the prepared dataset. Three classes were compared as binary, with one to the rest. Continuing, data from the first class were classified in opposition to the combined data of the other two and the second class in opposition to the first and third class. Finally, data from the third class were classified into the combined data from the first and second classes, as shown in Figure 15.

In the first part, the proposed CNN for binary vehicle classification (small vehicles to the rest of the vehicles) using the training dataset was trained. This training dataset was modified to a ratio of 1:1 (800 records for each class). The results from the test dataset are shown in Table 7.

In the second part, the proposed CNN for binary vehicle classification (SUV vehicles to the rest of the vehicles) using the training dataset was trained. This training dataset was modified to a ratio of 1:1 (800 records for each class). The results from the test dataset are shown in Table 8.

In the third part, the proposed CNN for binary vehicle classification (truck vehicles to the rest of the vehicles) using the training dataset was trained. This training dataset was modified to a ratio of 1:2 (400 records for the van/truck class, 800 images for the rest of the vehicles). The results from the test dataset are shown in Table 9.

An improved process by binary predicting three classes as one to the rest achieved valid classification on the test dataset of 70.8%, as shown in Table 10. Each row in the confusion matrix represents a test group for the class, and the columns represent the category. Highlighted values in the diagonal show properly classified vehicles.

Due to there being no information about vehicle speed, the wheelbase with axle configurations, and wheel sizes, the valid classification of 70.8% achieved is acceptable. These results are from one line of vertical FBG sensors with partially overpassing vehicles by one line of wheels. The speed and wheelbase similarities between the first and second classes created significant incorrect classifications, which can be reduced with a larger part of the dataset for the training of the designed CNN.

## 4. Discussion

The obtained results from the experimental platform, which consists of vertically oriented FBG sensor arrays, are presented. Due to the location of the testing platform on the two way access road into the university campus, because the sensor arrays are installed in the middle of this road, there was some limitation. More than 80% of recorded vehicles passed over the inbuilt sensors. Those vehicles were recorded only with one line of wheels without measurement by the horizontally oriented FBG sensors. A minimum of two lines of sensors is necessary for wheelbase distance determination and vehicle speed measuring. We focused on the classification of passing vehicles only from one line of vertical FBG sensors. The proposed neural network was capable of separating trucks from other vehicles with an accuracy of 94.9%. To classify three different classes, an accuracy of 70.8% was achieved. Based on experimental results, extending the sensor arrays is recommended.

**Figure 16:**
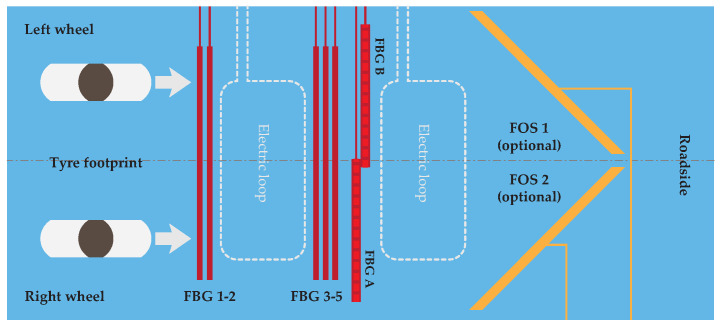
Proposed experimental platform with Fiber Optic Sensors (FOS) with 45° orientations (orange color).

The approach that solved the width of the vehicle’s wheels is based on horizontal fiber optic sensors with a 45° orientation in the vehicle’s direction over the test platform for the left and right side of the vehicle, separated as shown in Figure 16. This solution is limited by vehicle speed due to the double bridge construction over the road. Another technological approach, which is already widely used, is based on bending plates installed in a concrete block of the road. These sensors could be separated for the left and right side of the vehicle or be combined for weighing the whole vehicle’s axle. With these realizations, there is no need to know the wheel width, because there is a whole contact area of the wheel with road sensors.

In order to gain significant improvements of these results, it would be necessary to extend the sensor arrays to the full width of the road. An alternative solution will be a change from two way road management to one way.

## Figures and Tables

**Table 1:** Image dataset.

Vehicle Type	Class	Train	Test
Hatchback	1	650	428
Sedan/Combo	2	650	384
SUV	3	650	304
MPV/Minivan	4	650	227
Van/Truck	5	650	376

**Table 2:** Outputs from CNNs for image classification.

	AlexNet	GoogLeNet	ResNet-50
Achieved train validation	99.79%	90.67%	91.30%
Achieved test validation	90.2%	90.8%	89.2%

**Table 3:** Design of CNN for vehicle classification.

Layers	Parameters	Number of CFs ^🟉^
Input	600 × 5 × 1	
Conv2D + ReLU	300 × 4	128
Conv2D + ReLU	100 × 4	64
Conv2D + ReLU	100 × 4	32
MaxPool2D	2 × 1	
Conv2D + ReLU	50 × 2	24
FC + Softmax	2, 3 or 5	
ClassOutput	2, 3 or 5	

^🟉^ Convolution Filter (CF).

**Table 4:** Results from the test part of the dataset from the CNN for vehicle classification. Final results for each class on the main diagonal in confusion matrix (highlighted as bold) are shown.

Class	1	2	3	4	5	Valid
**1**	**9.8%**	1.7%	4.7%	3.3%	0.3%	49.6%
**2**	17.8%	**25.8%**	19.5%	9.8%	1.8%	12.8%
**3**	2.5%	0.8%	**8.5%**	3.0%	0.4%	56.3%
**4**	1.4%	0.4%	2.3%	**1.7%**	0.5%	26.8%
**5**	0.1%	0.1%	0.3%	0.6%	**1.8%**	62%
**Overall**						**28.9%**

**Table 5:** Results from the test part of the dataset from the CNN for vehicle classification reduced to 3 classes. Final results for each class on the main diagonal in confusion matrix (highlighted as bold) are shown.

Class	1	2	3	Valid
**1**	**38.9%**	10.9%	2.5%	74.3%
**2**	18.8%	**15.2%**	6.2%	37.8%
**3**	1.1%	0.5%	**5.9%**	78.9%
**Overall**				**60%**

**Table 6:** Results from the test part of the dataset from the CNN for vehicle classification reduced to 2 classes. Final results for each class on the main diagonal in confusion matrix (highlighted as bold) are shown.

Class	1	3	Valid
**1**	**83.2%**	4.2%	95.1%
**3**	3.0%	**9.6%**	76.1%
**Overall**			**92.7%**

**Table 7:** Results from the test part of the dataset from the CNN for vehicle binary classification. Final results for each class on the main diagonal in confusion matrix (highlighted as bold) are shown.

Class	1	2,3	Valid
**1**	**42.7%**	9.7%	81.6%
**2,3**	16.9%	**30.8%**	64.6%
**Overall**			**73.5%**

**Table 8:** Results from the test part of the dataset from the CNN for vehicle binary classification. Final results for each class on the main diagonal in confusion matrix (highlighted as bold) are shown.

Class	2	1,3	Valid
**2**	**21.7%**	18.3%	54.2%
**1,3**	10.9%	**49%**	81.8%
**Overall**			**70.7%**

**Table 9:** Results from the test part of the dataset from the CNN for vehicle binary classification. Final results for each class on the main diagonal in confusion matrix (highlighted as bold) are shown.

Class	3	1,2	Valid
**3**	**4.5%**	3.1%	59.2%
**1,2**	2%	**90.5%**	97.8%
**Overall**			**94.9%**

**Table 10:** Confusion matrix of classified vehicles. Final results for each class on the main diagonal in confusion matrix (highlighted as bold) are shown.

Class	1	2	3	Valid
**1**	**40.9%**	10.9%	0.5%	78.1%
**2**	13.8%	**25.8%**	0.5%	64.3%
**3**	1.3%	2.1%	**4.1%**	54.9%
**Overall**				**70.8%**

## Abbreviations

The following abbreviations are used in this manuscript:

2D	Two-Dimensional
3D	Three-Dimensional
Avg	Average
CCN	Convolutional Neural Network
CCTV	Closed-Circuit Television
CF	Convolution Filter
Conv2D	Two-Dimensional Convolution layer
Conv2D R	Conv2D Reduce
COST	European Cooperation in Science and Technology
DAG	Directed Acyclic Graph
FBG	Fiber Bragg Grating
FC	Fully Connected
FOS	Fiber Optic Sensor
GPU	Graphic Processor Unit
HO	Horizontally Oriented
IDE	Integrated Development Environment
IoT	Internet of Things
MPV	Multi-Purpose Vehicle
NoR	Nothing-on-Road
px	pixel(s)
ReLU	Rectified Linear Unit
SUV	Sports Utility Vehicles
TIFF	Tagged Image File Format
VO	Vertically Oriented
